# Intraarticular treatment with integrin α10β1-selected mesenchymal stem cells affects microRNA expression in experimental post-traumatic osteoarthritis in horses

**DOI:** 10.3389/fvets.2024.1374681

**Published:** 2024-03-26

**Authors:** Camilla Andersen, Marie Walters, Louise Bundgaard, Lise Charlotte Berg, Lucienne Angela Vonk, Evy Lundgren-Åkerlund, Betina Lyngfeldt Henriksen, Casper Lindegaard, Kerstin Skovgaard, Stine Jacobsen

**Affiliations:** ^1^Department of Veterinary Clinical Sciences, Faculty of Health and Medical Sciences, University of Copenhagen, Taastrup, Denmark; ^2^Xintela AB, Lund, Sweden; ^3^Department of Biotechnology and Biomedicine, Technical University of Denmark, Lyngby, Denmark

**Keywords:** microRNA, mesenchymal stem cells, osteoarthritis, cartilage, horse

## Abstract

Osteoarthritis (OA) remains a major cause of lameness in horses, which leads to lost days of training and early retirement. Still, the underlying pathological processes are poorly understood. MicroRNAs (miRNAs) are small non-coding RNAs that serve as regulators of many biological processes including OA. Analysis of miRNA expression in diseased joint tissues such as cartilage and synovial membrane may help to elucidate OA pathology. Since integrin α10β1-selected mesenchymal stem cell (integrin α10-MSC) have shown mitigating effect on equine OA we here investigated the effect of integrin α10-MSCs on miRNA expression. Cartilage and synovial membrane was harvested from the middle carpal joint of horses with experimentally induced, untreated OA, horses with experimentally induced OA treated with allogeneic adipose-derived MSCs selected for the marker integrin α10-MSCs, and from healthy control joints. miRNA expression in cartilage and synovial membrane was established by quantifying 70 pre-determined miRNAs by qPCR. Differential expression of the miRNAs was evaluated by comparing untreated OA and control, untreated OA and MSC-treated OA, and joints with high and low pathology score. A total of 60 miRNAs were successfully quantified in the cartilage samples and 55 miRNAs were quantified in the synovial membrane samples. In cartilage, miR-146a, miR-150 and miR-409 had significantly higher expression in untreated OA joints than in control joints. Expression of miR-125a-3p, miR-150, miR-200c, and miR-499-5p was significantly reduced in cartilage from MSC-treated OA joints compared to the untreated OA joints. Expression of miR-139-5p, miR-150, miR-182-5p, miR-200a, miR-378, miR-409-3p, and miR-7177b in articular cartilage reflected pathology score. Several of these miRNAs are known from research in human patients with OA and from murine OA models. Our study shows that these miRNAs are also differentially expressed in experimental equine OA, and that expression depends on OA severity. Moreover, MSC treatment, which resulted in less severe OA, also affected miRNA expression in cartilage.

## Introduction

Lameness in horses is the most important reason for lost days of training and early retirement, and may lead to euthanasia (1–4). Osteoarthritis (OA) accounts for up to 60% of all cases of equine lameness (5). OA is a complex heterogenous disease with multiple causative factors including trauma or overuse, poor conformation of the joints, and advancing age (4). OA affects the entire joint and is characterized by inflammation of the synovium, thickening of the joint capsule, fraying of the ligaments, subchondral bone sclerosis, and irreversible destruction of the articular hyaline cartilage (6–8). The underlying pathophysiology of OA is poorly understood, and equine OA is mainly diagnosed based on radiography, computed tomography, or magnetic resonance imaging, which will only detect changes when the disease is progressed and osteophytes and significant cartilage damage has already occurred. As articular cartilage has limited capacity to heal, these changes cannot be reversed (9, 10). Therefore, there is demand for early diagnostics, improved therapeutics and for continued research into OA pathology.

Mesenchymal stem cell (MSC) treatment is attracting increasing attention for treatment of OA, and intra-articular MSC treatment has shown beneficial effects on OA in studies of experimentally induced (11–18) and naturally occurring equine OA (19–22). MSCs have the ability to differentiate into chondrocytes *in vitro* (23, 24), and have been shown to regenerate cartilage in vivo (25). MSCs are also known to release immunomodulatory factors, which decrease inflammation and promote healing, which contribute to their disease modifying effects (26). These secreted factors also include miRNAs. It has been shown that MSC derived extracellular vesicles (EVs) containing the miRNAs let-7a-5p, miR-100-5p, miR-122-5p, miR-148a-3p, and miR-486-5p, promoted macrophage M2 polarization and anti-inflammatory IL-10 release *in vitro* and exerted a chondroprotective effect in an OA-model in rats *in vivo* (27). Another study found a variety of miRNAs derived from MSCs *in vitro*, including the let-7 family, miR-29b, miR-122, miR-125b, and miR-148a. Both miR-29b and miR-148a are known to promote the differentiation of chondrocytes and the secretion of proteoglycans and type II collagen (28), while miR-125b decreases extracellular matrix (ECM) degradation through down-regulation of A Disintegrin and Metalloproteinase with Thrombospondin motifs-4 (ADAMTS-4) (29). MSC preparations from different sources are known to consist of a heterogeneous mix of cells, and selection of the MSC populations for therapeutic use has been suggested to result in superior clinical effect (30–32). Using the MSC marker integrin α10β1 for selection has been shown to result in consistent and homogeneous MSC preparations (33, 34). MSCs selected for the expression of integrin α10β1 (integrin α10-MSCs) showed better adherence to damaged cartilage and exposed subchondral bone *in explants*, increased type II collagen deposition after chondrogenic differentiation in vitro, and higher secretion of the immunomodulatory factor prostaglandin E2 after stimulation *in vitro* compared to unselected MSCs (34). The homing ability and regenerative capacity of integrin α10-MSCs was demonstrated in a rabbit model of cartilage damage (25), and treatment with integrin α10-MSCs has previously been shown to mitigate the progression of OA in an equine model (13). The current study used cartilage and synovial membrane samples from a previous study, in which a clinical effect of integrin α10-MSC treatment was seen both on clinical presentation, post mortem evaluation of articular cartilage and on synovial fluid prostaglandin E2 and interleukin-10 (35).

MicroRNAs (miRNAs) are small non-coding RNAs that serve as regulators of many biological processes, including embryologic development, proliferation, apoptosis, and cell metabolism (36). MiRNAs regulate gene expression on a post-translational level by guiding the RNA-induced silencing complex (RISC) to a target messenger RNA (mRNA), resulting in degradation or translational repression. Therefore, altered miRNA expression can alter the translational landscape within a cell, facilitating or repressing multiple disease pathways (37). Multiple miRNAs have been associated with processes involved in OA pathogenesis and cartilage aging (38), and a number of miRNAs have been identified to be differentially expressed in synovium and cartilage from healthy and diseased joints including let-7a, let-7e, miR-10a, miR-23b, miR-26a, miR-27, miR-99a, miR-125, miR-139, miR-140, miR-146a, miR-151–5p, miR-200c, and miR-378 (39–49) and many more. Understanding miRNA regulation is complex, as each miRNA targets several hundreds mRNAs and each mRNA can be targeted by a wide range of miRNAs (50). MiRNAs exhibit greater resistance to ribonucleases, extreme temperatures, and pH variations when compared to longer RNA molecules.

In particular, circulating miRNAs, released from cells into the bloodstream or cerebrospinal fluid, have demonstrated remarkable stability attributed to their transportation within exosomes or microvesicles, or their association with protein complexes. The high stability points toward miRNAs as promising candidates for future clinical biomarkers of osteoarthritis in horses (51–54). The aim of this study was to identify differentially expressed miRNAs in the articular cartilage and synovial membrane of horses with experimentally induced OA and to assess effect of intra-articular treatment with integrin α10-MSC. The study design involved samples from healthy control joints and joints with experimentally induced OA joints, which were either untreated or integrin α10-MSC-treated, in order to investigate possible epigenetic mechanisms of action behind OA development and MSC-treatment in horses.

## Materials and methods

### Horses and study design

This study used cartilage and synovial membrane from the middle carpal joint (MCJ) of two groups of horses with experimentally induced post traumatic osteoarthritis either treated with integrin α10-MSCs or left untreated and from the control joint from the untreated group. A total of seventeen healthy Standardbred trotters aged 3–7 years (mean: 4.7 years; median 4 years), weight range 396–535 kg (mean: 472 kg; median 470 kg), 15 mares and two geldings were included in the study. There were eight horses in the MSC-treated group and nine horses in the untreated group. The horses underwent repeated clinical assessments and had blood and synovial fluid sampled sequentially throughout the 70-day study period for other purposes as described in a previous publication by our group (35). The horses were euthanized at study termination on day 70 with pentobarbital (Euthasol^®^ Vet, Le Vet B.V., Oudewater, NL) 140 mg/kg, after sedation with detomidine (Domosedan^®^ Vet, Orion Corporation, Espoo, Finland) 1 mg/100 kg and butorphanol 3 mg/100 kg (Dolorex^®^, Ag Marin Pharmaceuticals, United States). The samples obtained post-mortem were analyzed in the present study. For OA-induction surgery, the horses were premedicated with a combination of romifidine 6 mg/100 kg (Sedivet^®^Vet, Boehringer Ingelheim Vetmedica, Missouri, United States), acepromazine 3 mg/100 kg (Plegicil^®^ Vet, Boehringer Ingelheim Vetmedica, Missouri, United States), atropine sulfate 0.5 mg/100 kg (Atropin, Aguettant Ltd., Bristol, United Kingdom), and butorphanol 3 mg/100 kg (Dolorex^®^, Ag Marin Pharmaceuticals, United States). Anesthesia was induced with ketamine 2.5 mg/kg (Ketador^®^ Vet, Richter Pharma AG, Oberosterreich, Austria) and midazolam 4 mg/100 kg (Midazolam “Accord,” Accord-UK Ltd., Barnstaple, United Kingdom). The horses were placed in dorsal recumbency and anesthesia maintained with isoflourane (Vetflurane^®^, Virbac, Carros, France). Perioperatively the horses received flunixin meglumine 1.1 mg/kg (Finadyne, MSD Animal Health, New Jersey, United States), penicillin 22,000 IU/kg (Benzylpenicillin PanPharma, Brancaster Pharma, Surrey, United Kingdom), and gentamicin 6.6 mg/kg (Genta-Equine, Dechra Veterinary Products, Shrewsbury, United Kingdom).

### Osteoarthritis model

Osteoarthritis was induced through the carpal osteochondral fragment-exercise model (17, 55–62). OA was induced in the left MCJ of all horses and the right MCJ was sham operated and served as a control joint. In the right carpus an osteochondral “chip” fracture was made with an 8 mm curved osteotome in the dorsal margin of the third facet of the distal surface of the radial carpal bone at the level of the medial plica. The fragment remained attached to the plica. The debris was not flushed from the joint.

The horses were stall rested for the first 14 days after surgery, interrupted by shorter daily periods of hand walking from day 2. Treadmill exercise was initiated on day 14 after surgery. The horses were exercised 5 days a week for 8 weeks through the following program: 2 min slow trot 16–19 km/h (4.4–5.3 m/s). 2 min fast trot 32km/h (9 m/s). 2 min slow trot 16–19 km/h (4.4–5.3 m/s). From day 14 the horses were also allowed free pasture-exercise every day.

### Integrin α10-MSC treatment

Allogeneic adipose equine MSCs were isolated from a 7-year-old male horse. The MSCs were culture expanded until passage 3 and selected for a high expression of integrin α10β1 were selected as previously described (35). On day 18 the horses in the treatment group were treated with 2 × 10^7^ equine allogeneic adipose tissue-derived and integrin α10β1 selected mesenchymal stem cells in 4 ml DMSO cryopreservation medium (Cryostor, BioLife Solutions). The α10-MSCs were thawed in a water bath at 37°C, aspirated into a syringe through a 14G canula at a slow pace and injected into the MCJ of the OA leg through a 20G canula over a minimum of 10 s.

### Postmortem macroscopic pathology evaluation

Shortly after euthanasia, the MCJs were opened by careful sharp dissection and photographed in detail for later blinded assessment of macroscopic pathology to assess the development of OA and the clinical effect of MSC treatment on experimentally induced OA (35). Macroscopic pathology was scored from detailed photographs by observers blinded to treatment group using a detailed score developed by our group (63). In brief, each carpal bone in the MCJ was assessed separately according to erosion severity and extent of erosions, which were multiplied resulting in a total cartilage erosion score. The synovial membrane was scored separately. These results were published previously (35). Here, they only serve to describe the groups.

Macroscopic pathology scoring showed that both the treated and the untreated OA joints had developed generalized cartilage erosions. There was more macroscopic cartilage pathology in the untreated OA joints (mean pathology score=25.7; CI = 13.0–38.4) compared to the control joints (mean pathology score = 9.6; CI = 4.5–14.7), although this difference was not significant (*p* = 0.0629). There was significantly more macroscopic cartilage pathology (*p* = 0.0491) in the untreated OA joints compared to the MSC-treated OA joints (mean pathology score = 11.0; CI = 4.1–17.9) (35).

There was no difference in synovial membrane pathology score between the untreated OA joint and control joint (*p* = 0.128) or between the MSC-treated and untreated OA joints (*p* = 0.91) (35).

### Tissue sampling

Tissue samples were collected shortly after euthanasia. Synovial membrane samples were collected using sharp scissors. Samples were taken and pooled from the entire dorsal aspect of the middle carpal joint. Cartilage samples were shaved of the third facet of the radial carpal bone with a scalpel blade. This included cartilage adjacent to the surgically created fragment, but with a minimal distance to the fragment of 3–4 mm. Both tissues were placed in cryotubes containing RNAlater (Qiagen) and kept at 5°C overnight and stored at −20°C until further analyses.

### Target miRNA and primer design

Relative miRNA expression in cartilage and synovial membrane was established by quantifying the following 70 miRNAs: Let-7a, Let-7c, let-7d, Let-7e, Let-7f, let-7g, miR-10a, miR-10b, miR-19a, miR-20a, miR-23a, miR-26a, miR-27a miR-27b, miR-28-3p, miR-28-5p, miR-29a, miR-29b, miR-30b, miR-30c, miR-30d, miR-98, miR-99a, miR-101, miR-122, miR-125a-3p, miR-125a-5p, miR-139-3p, miR-139-5p, miR-140-3p, miR-140-5p, miR-146a, miR-148a-3p, miR-148b-3p, miR-148b-5p, miR-150, miR-151-5p, miR-155, miR-182-5p, miRNA-183, miR-184, miR-186, miR-192, miR-195, miR-196b-3p, miR-199b-3p, miR-199b-5p, miR-200a, miR-200b, miR-200c, miR-214-3p, miR-215, miR-296, miR-329b, miR-340-3p, miR-340-5p, miR-342-3p, miR-342-5p, miR-374a, miR-378, miR-409-3p, miR-499-3p, miR-499-5p, miR-676, miR-872, miR-1307, miR-1388, miR-1839, miR-7177b, and miR-8992 (A list of miRNAs and specific primer sequences are included in Supplementary Table S1). These 70 miRNAs have been selected based on literature and smallRNA sequencing in equine synovial fluid in a previous study (submitted for publication). Primers were designed based on the principles described by Balcells et al. (64). Primers were designed using miRprimerdesign3 (65) and synthesized by Sigma-Aldrich (Sigma–Aldrich, Brøndby, Denmark).

### Microfluidic high throughput RT-qPCR (Fluidigm)

#### RNA extraction

Cartilage and synovial membrane tissue samples were homogenized in 1 ml QIAzol LysisReagent (Qiagen) using a gentleMACS^TM^ Dissociator (Milteny Biotec, GmbH, Bergisch Gladbach, Germany). Total RNA was extracted using miRNeasyMini Kit (Qiagen), and all samples underwent on-column DNase digestion with RNase free DNase sets (Qiagen), according to the manufacturer’s instructions. RNA purity and concentration was determined by spectrophotometry (Nano Drop ND-1000, NanoDrop Technologies, Saveen and Werner AB, Limhamn, Sweden), and RNA integrity was measured on an Agilent 2100Bioanalyzer (Agilent Technologies, Nærum, Denmark) using the RNA 6000 Nano Kit. Minimum RNA integrity number (RIN) accepted was 6.5 and average RIN was over 8. Both miRNA and the assay used for detection (microfluidic qPCR) are generating highly reproducible data, even for more degraded samples (data not shown).

#### cDNA synthesis, pre-amplification, and exonuclease treatment

RNA was converted into single stranded cDNA by reverse transcription in two consecutive steps, where three separate cDNA syntheses (technical replicates) were performed for each RNA sample. Hundred nanogram total RNA was mixed with a Master mix containing 1 μL 10 x reaction buffer polyA (New England Biolabs, Ipswich, MA, United States), 1 μL 1 mM ATP (New England Biolabs, Ipswich, MA, United States), 1 μL 1 mM dNTP mix (Sigma–Aldrich, Brøndby, Denmark), 1 μL 10 μM RT-primer (5′ caggtccagtttttttttttttttvn 3′) (Sigma–Aldrich, Brøndby, Denmark), 0.5 μL reverse transcriptase 200,000 U/mL (New England Biolabs, Ipswich, MA, United States) and 0.2 μL PolyA polymerase 5,000 U/mL (New England Biolabs, Ipswich, MA, United States). Master mix and RNA were incubated using a thermocycler (TProffessional TRIO 3x48, Fisher Scientific, Denmark, Slangerup, Denmark), and reverse transcription occurred at 42°C for 60 min prior to 5 min inactivation at 95°C. Three non-reverse transcriptase controls (-RT) were included during the procedure for later use as control samples.

Samples were pre-amplified and exonuclease treated using the following protocol in order to ensure sufficient amount of DNA for quantification and to degrade unincorporated primers. Stocks of 200 nM primer mix were prepared containing equal amounts of the selected primer pairs and mixed with low EDTA TE-buffer (VWR-Bie & Berntsen, Herlev, Denmark). Afterwards 3 μL TaqMan PreAmp Master Mix (Applied Biosystems, Foster City, CA, United States), 2 μL low EDTA TE-buffer, 2.5 μL 200 nM primer mix, and 2.5 μL cDNA were mixed and incubated in a thermocycler for 16 cycles (95°C for 10 min followed by 15 s at 95°C and 4 min at 60°C). Pre-amplified cDNA was subsequently treated with 4 U/μL exonuclease (New England Biolabs, Ipswich, MA, United States) and incubated at 37°C for 30 min followed by 80°C for 15 min. Finally, the samples were diluted 1:10 in low EDTA TE Buffer and stored at −20°C until microfluidic qPCR was performed.

An aliquot of undiluted pre-amplified cDNA was saved for preparation of dilution series.

#### Microfluidic qPCR

High throughput microfluidic qPCR was performed using the 96.96 dynamic array integrated fluidic circuits chip (Fluidigm, San Francisco, CA, United States) combining 96 samples with 96 primer sets in 9216 separate simultaneous qPCR reactions.

An assay master mix was prepared and consisted of 3 μL 2X Assay Loading Reagent (Fluigdim, San Francisco, CA, United States) and 3 μL 10 μM forward and reverse primer.

Then a Pre-sample mix was made of 3 μL TaqMan Gene Expression Master mix (Applied Biosystems, Foster City, CA, United States), 0.3 μL 20X DNA Binding Dye Sample Loading Reagent (Fluidigm, San Francisco, CA, United States), 0.3μL 20 X EvaGreen (Biotium; VWR- Bie & Berntsen, Herlev Denmark), 0.9 μL low TE-buffer and 1.5 μL pre-amplified cDNA.

Samples and primers were loaded in the microfluidic chip according to the manufacturer’s instructions, and the BioMark (Fluidigm, San Francisco, CA, United States) was used to perform the microfluidic qPCR cDNA technical triplicates, no template control, -RT samples, amplification curves, melting curves, and dilution curves all served as different quality controls points for each process and were used in the subsequent data preprocessing. Data were handled by the Fluidigm Real-Time PCR Analysis software 3.0.2 (Fluidigm, San Francisco, CA, United States).

### Data processing and statistical analyses

Data was corrected for PCR efficiency by standard curves, efficiency between 85 and 115% were accepted before correction. Expression values were normalized using global normalization in order to reduce non-biological variation between samples. To ensure data reproducibility of each primer set, cDNA technical replicates were compared and excluded if the deviation was too high (>1.0 Cq between triple determinations). Tissue samples and/or targets with more than 15% of values with more than 1.0 Cq between triple determinations were excluded. cDNA technical replicates were averaged for each miRNA. miRNA expression data never reaching the fluorescence threshold (1.09% of samples for cartilage and 0.0% for SM) were assigned the highest Cq measured (= lowest expression) for the miRNA in question in any sample + 1. Subsequently, expression data (Cq values) were transformed into relative quantities (Rq) and log2 transformed prior to statistical analysis.

Normality of data was assessed using Shapiro-wilks test, histograms and QQ-plots. Gross pathology scores of the untreated control joints and the treated and untreated OA joints were compared using Wilcoxon Exact Test analyzed with R.

Differential expression of the various miRNA Rq was compared between the following groups: untreated OA joint versus untreated control joint, and untreated OA joint versus MSC-treated OA joint. Because of some variance in macroscopic pathology in all groups (untreated control joint, untreated OA joint and MSC-treated OA joint), meaning that we observed joints of both high and low pathology score in all groups, we also compared all joints with a pathology score above versus below 10 across groups, to evaluate how the severity of cartilage erosions impacted the miRNA expression and separate these effects from the effect caused solely by MSC-treatment. The fold change (FC) between groups was calculated for each of the miRNAs using Microsoft Excel. miRNAs were considered differentially expressed when the level of significance was below 0.05 and FC was at least 50% (<0.5 or >1.5).

Principal component analysis (PCA) and heatmap were created in RStudio (version 4.2.2), using the ggplot2 and pheatmap package. The PCAs were based on log2 transformed relative quantities, and the heatmap was based on autoscaled log2 transformed relative quantities.

## Results

### miRNAs in the cartilage were differentially expressed between groups

**Figure 1:**
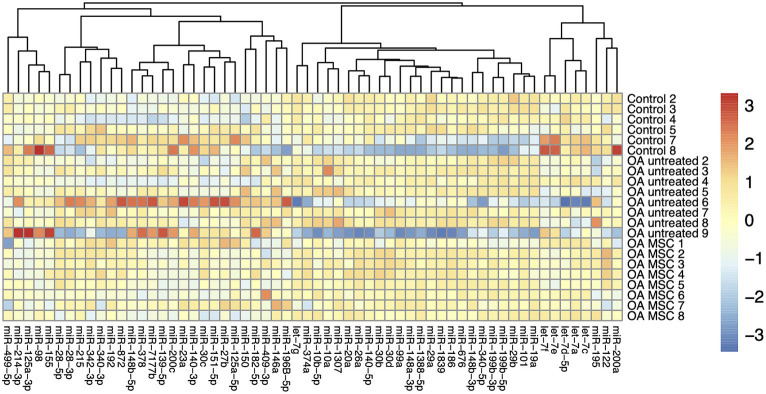
Heatmap of autoscaled log2 transformed relative quantities of miRNA expression in cartilage of the three different groups; control, OA untreated, and OA MSC. Each individual horse has been provided with a number from 1 to 9. The miRNAs are visualized vertically, while the samples are depicted horizontally. MiRNA with similar expression profiles are clustered closer together.

A total of 60 out of the 70 miRNAs were successfully identified in the cartilage samples (Supplementary File S2) The heatmap did not reveal any shared expression signature among the three treatment groups based on the 60 miRNAs (Figure 1). Three out of the 60 miRNAs were differentially expressed in the cartilage between the untreated OA joints and untreated control joints (miR-146a; miR-150; miR-409-3p) (Table 1). Four out of the 60 miRNAs were differentially expressed in the cartilage between the untreated OA joints and the MSC-treated OA joints (miR-125a-3p; miR-150; miR-200c; miR-499-5p), while additionally 4 miRNAs were likely to be upregulated (miR-139a-5p; miR-378; miR-409 and miR-7177b), although the expression difference did not reach significance (*p* > 0.05, but < 0.09) (Table 1). Seven out of the 60 miRNAs were differentially expressed in the cartilage between joints with a pathology score above and below 10, respectively (miR-139-5p; miR-150; miR-182-5p; miR-200a; miR-378; miR-409-3p; miR-7177b), and one miRNA with a significant but only borderline differentially expressed (miR-125a-5p; *p* = 0.041; FC = 1.41) (Table 1).

**Table 1:** miRNAs differentially expressed in articular cartilage between groups.

	Untreated OA versus untreated control cartilage		Untreated versus MSC-treated OA cartilage		High versus low pathology score		Interpretation
miRNA	FC	*p*-value	FC	*p*-value	FC	*p*-value	
miR-125a-3p			8.99	0.037*			**↓** in MSC-treated joints
miR-125a-5p					1.41	0.041*	**↑** with high pathology score
miR-139-5p			5.3	0.062	3.976	0.033*	**↓** in MSC-treated joints and**↑** with high pathology score
miR-146a	3.37	<0.001***					**↑** in OA joints compared to controls
miR-150	2.39	0.032*	2.83	0.019*	2.9	0.003**	**↓** in MSC-treated joints,**↑** in OA joints compared to controls and with high pathology score
miR-182-5p					3.46	<0.01**	**↑** with high pathology score
miR-200a					0.37	0.046*	**↓** with high pathology score
miR-200c			3.35	0.048*			**↓** in MSC-treated joints
miR-378			3.16	0.077	2.89	0.031*	**↓** in MSC-treated joints and**↑** with high pathology score
miR-409-3p	2.3	0.024*	1.46	0.089	2.21	0.013*	**↓** in MSC-treated joints,**↑** in OA joints compared to controls and with high pathology score
miR-499-5p			2.19	0.045*			**↓** in MSC-treated joints
miR-7177b			3.80	0.067	3.51	0.016*	**↓** in MSC-treated joints and**↑** with high pathology score

FC, fold change; OA, osteoarthritis. *p < 0.05; **p < 0.01; ***p < 0.001.

Microfluidic qPCR failed to quantify 9 out of the 70 miRNAs in all of the cartilage and synovial membrane samples (miR-27A; miR-139-3p; miR-183; miR-184; miR-200b-3p; miR-296; miR-329b-3p; miR-499-3p; and miR-8992). In addition, miR-342-5p was not present in any of the cartilage samples, but was found in the synovial membrane.

### There was no difference between groups for miRNA expression in synovial membrane

We were able to quantify 55 out of the 70 miRNAs in the synovial membrane samples from untreated control joints and untreated and treated OA joints and MSC-treated OA joints. In contrast to the cartilage samples, miR-182-5p; miR-200a; miR-215; miR-374a; miR-1388-5p and miR-7177b were not present in synovial membrane, while miR-342-5p was identified in synovial membrane but not in cartilage. None of the 55 miRNAs were differentially expressed in the synovial membrane between untreated OA- and untreated control joints, between untreated- and MSC-treated OA joints, or between joints with high and low pathology score.

### Tissue specific miRNA expression signatures in cartilage and synovial membrane

**Figure 2:**
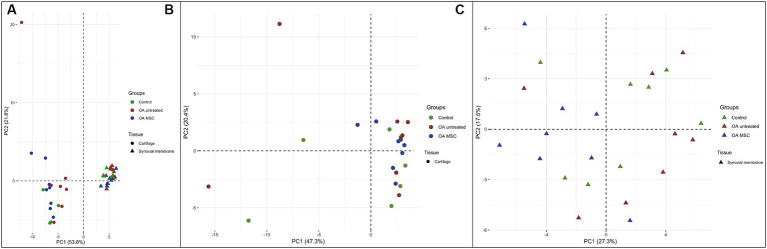
Principal component analysis (PCA) of the three different groups; control (green), OA untreated (red) and OA MSC (blue) in the two different tissues; cartilage (dot) and synovial membrane (triangle) using two principal components (PCs) as coordinate axes plotted against miRNA expression levels of the 54 different miRNAs quantified in both cartilage and synovial membrane. **(A)** Samples from cartilage and synovial membrane form two separate clusters, meaning the two clusters display diverse miRNA expression profiles. **(B)** Samples from only cartilage and **(C)** samples from only synovial membrane do not form separate clusters between groups.

Fifty-four miRNAs were identified in both cartilage and synovial membrane, of which 31 were significantly differentially expressed between the two tissues. Cartilage and synovial membrane had different miRNA expression profiles based on a PCA, as the two tissues formed two separate clusters (Figure 2). The different samples of the synovial membrane clustered more tightly than cartilage, indicating that the miRNA expression profile were more uniform in synovial membrane than in cartilage in our model. The 31 differentially expressed miRNAs were: miR-10a; miR-10b; miR-19a; miR-20a; miR-27b; miR-28-5p; miR-29b; miR-99a; miR-101; miR-122; miR-125a-3p; miR-139-5p; miR-140-3p; miR-140-5p; miR-146a; miR-148a-3p; miR-148b-3p; miR-150; miR-155; miR-182; miR-192; miR-199b-3p; miR-199-5p; miR-200c; miR-340-5p; miR-342-3p; miR-378; miR-409-3p; miR-499-5p; miR-676; and miR-1307.

## Discussion

Differential expression of several miRNAs has been associated with OA in humans and laboratory animal species (39–49). We were able to quantify 60 and 55 miRNAs in articular cartilage and synovial membrane, respectively, from horses with experimental OA. The combined analysis of all miRNAs investigated did not result in a clustering of the three analyzed groups; untreated OA, MSC treated OA, and control. However, several individual miRNAs in cartilage were differentially expressed between the three groups.

Three miRNAs were upregulated in cartilage from untreated OA joints compared to control joints (miR-146A; miR-150; miR-409-3p). Both miR-146a (36, 41, 66–69) and miR-150 (70, 71) are known to be related to OA, whereas miR-409-3p (72, 73) has been described to be involved in regulation of inflammation, which is a prominent part of the OA pathogenesis.

miR-146a is one of the most extensively studied miRNAs in relation to OA and inflammation. It has been found to have increased expression in cartilage of OA joints compared to controls in a number of previous studies in human patients (41, 66–69), and has therefore been suggested as a biomarker of OA. Bioinformatics analysis has demonstrated that miR-146a targets 159 proteins (67), including inflammatory inducers in the nuclear factor kappa B pathway and IL-1β, and factors involved in apoptosis or autophagy (67), which might have been ongoing at the time our horses were euthanized (day 70 after OA-induction). There was no correlation between pathology score and expression of miR-146, corresponding to findings by Yamasaki et al. (74), who showed that miR-146 had increased expression in OA cartilage compared to healthy cartilage, but with no correlation to the severity of pathological changes. Considering not only severity, but also chronicity, of OA may be important for characterizing the expression pattern of miRNAs on osteoarthritic cartilage. In humans, miR-146 was downregulated in cartilage of patients with late-stage OA compared to healthy cartilage (75), suggesting that miR-146a plays a role mainly in the early and intermediate stages of OA.

Two miRNAs are of particular interest based on our results. Expression of miR-409-3p and miR-150 seemed to reflect presence and severity of OA in our experimental setup; expression was higher in OA cartilage compared to control, higher in cartilage with high pathology score, and lower in OA cartilage after MSC treatment, which resulted in reduced OA severity (35).

miR-409-3p has not previously been described in OA or cartilage models. However, miR-409-3p has been shown to increase the pro-inflammatory response by inhibiting the anti-inflammatory suppressor of cytokine signaling 3 (SOCS3) in non-joint cell types *in vivo* and *in vitro* (72, 73).

SOCS3 is known to play a critical role intraarticularly by preventing cartilage loss in arthritic conditions (76–79), where it inhibits signaling pathways of pro-inflammatory cytokines and catabolic factors such as metalloproteinase-3 (MMP-3), MMP-13, interleukin-6 and inducible nitric oxide synthase (79, 80). While never described in OA before, miR-409-3p may be linked to presence and severity of the disease through its down-stream effects on SOCS3, and this warrants further investigation in future studies.

In our model of post-traumatic OA, miR-150 expression seemed to be negatively associated with OA, as expression was higher in cartilage of untreated OA joints compared to control joints and in joints with high pathology score and was reduced by MSC treatment in OA affected joints. This is in contrast to previous studies reporting downregulation of miR-150 in OA cartilage in human patients, in murine OA models and *in vitro* (70, 71, 81), and a study suggested a therapeutic effect of EVs containing miR-150 on OA (82–84). The reason for these diverging results is not clear but could be related to species differences or to variations in pathogenesis of different types of OA. Expression of miR-150 is increased in rheumatoid arthritis (85), a highly inflammatory type of arthritis, and the increased miR-150 expression in our model could potentially be related to a more severe inflammatory response than encountered in naturally occurring OA.

Expression of miRNAs was not only affected by the presence of OA, but also by the severity of cartilage pathology—with higher expression of the aforementioned two miRNAs (miR-409-3p and miR-150) as well as several others (miR-125a-5p, miR-139-5p, miR-182-5p, miR-378, and miR-7177b) in cartilage with high pathology score compared to cartilage with lower scores. In turn, miR-200a was downregulated in joints with more severe pathology. In our study there were joints with a high or a low pathology score in both the control group and in the OA groups. This could be related to the repeated synovial sampling, to the treadmill exercise program or to contralateral limb overuse caused by the compensatory movement pattern that occurs in lame horses (86, 87). Therefore, we investigated the differentially expressed miRNAs in joints with a pathology score above and below 10 across all groups.

Correlation between OA severity and expression of miRNAs has previously been demonstrated in humans (88, 89), thus suggesting that miRNAs could potentially help clinicians stage the disease.

Several of the miRNAs affected by pathology score were also downregulated in cartilage from MSC-treated OA joints compared to untreated OA joints, with miR-139-5p, miR-378, miR-409, and miR-7177b not quite reaching statistical significance (0.09 > *p* > 0.05). This might suggest that the downregulation in the MSC-treated joints was due to the reduced pathology in these joints rather than a direct effect of the MSCs.

A major aim of this study was to assess differences in miRNA expression in OA cartilage with and without MSC-treatment. MSC-therapy had chondroprotective effects in earlier studies involving models of equine OA (13, 14, 21), and this was confirmed in our study, as the MSC-treated OA group had significantly less severe cartilage pathology compared to the untreated OA group (35). In the present study, several miRNAs were downregulated in articular cartilage from the MSC-treated OA joints relative to the untreated OA joints. These changes can be directly related to the MSCs or be due to the reduced OA in treated joints. It is well-described that MSCs exert immunomodulatory effects that could affect expression of miRNAs and thereby influence progression of OA (26).

The most prominent difference between MSC-treated and non-treated OA joints was the differential expression of miR-125a-3p, which was 9 times lower in MSC-treated OA cartilage than non-treated OA cartilage. miR-125a-3p has not previously been described in OA or cartilage, but has been shown to affect osteogenic and adipogenic differentiation of stem cells (90, 91). Therefore, the observed lower expression of miR-125a-3p in MSC-treated OA cartilage could be associated with MSC differentiation rather than reduction in OA.

In contrast to cartilage, expression of miRNAs in synovial membrane was not affected by OA, severity of pathological changes, or treatment with MSCs. Comparable results were obtained in a murine model of post-traumatic OA, where none of 559 identified miRNAs were differentially expressed in synovial membrane from sham operated and experimental OA (destabilization of the medial meniscus) groups (92). The authors provided several potential explanations for this finding, including timing of sampling and the focal nature of the synovial response in OA. Such factors may also have affected the results of the present study. Synovitis is generally considered to occur in the early stages of OA (8) and sampling at day 70 after induction of OA may have been too late in the course of disease to detect differences in synovial membrane inflammation between groups. We have previously reported from this animal experiment that histology scores in synovial membranes from MSC-treated OA-affected joints did not differ from non-treated OA-affected joints (35). Also, the site of tissue sampling may have influenced the obtained results. Our study design encompassed weekly arthrocenteses (35, 93), which may have affected the results. Synovial membrane was sampled from the entire dorsal aspect of the joint, which is where arthrocenteses had been performed. Repeated arthrocentesis has been shown to causes joint inflammation with an increase in nitric oxide, prostaglandin E2, glycosaminoglycan and metalloproteinases concentrations in synovial fluid (94, 95). Therefore, synovial membrane inflammation caused by arthrocentesis may have masked differences between OA, control and MSC-treated joints in our study. Obviously, the lack of differential expression may also be a pathological feature of OA. Increased expression of miR-146a and miR-150 as well as other miRNAs has been demonstrated in the highly inflammatory and destructive rheumatoid arthritis (83, 84), but synovitis in OA is much less severe and more variable (8), which may explain less pronounced changes in synovial membrane miRNA expression.

Currently there are only few studies reporting expression of miRNAs in experimental and naturally occurring equine OA (54, 96–98). There is no consistency in the miRNAs identified as being differentially expressed in the studies, and it is therefore impossible at this stage to speculate about the validity of our or other experimental models in mimicking changes occurring in spontaneous equine OA.

## Conclusion

We were able to quantify 60 miRNAs in articular cartilage and 55 miRNAs in synovial membrane out of our 70 miRNA panel.

In cartilage, miR-146a, miR-150, and miR-409 had significantly higher expression in untreated OA joints than in control joints. Expression of miR-125a-3p, miR-150, miR-200c, and miR-499-5p was significantly reduced in cartilage from MSC-treated OA joints compared to the untreated OA joints. Expression of miR-139-5p, miR-150, miR-182-5p, miR-200a, miR-378, miR-409-3p, and miR-7177b in articular catilage reflected pathology score. In contrast, none of the miRNAs in synovial membrane showed differential expression between groups.

Most of the identified miRNAs have been described in OA and/or inflammation in other species than the horse. Our results thus expands the very limited knowledge on the miRNA landscape in equine OA by suggesting that these miRNAs play a role in OA in this species. Moreover, our findings suggest that MSC-treatment may have a direct or indirect (through reduction in OA severity) effect on miRNA expression in post-traumatic OA. Taken together, our results suggest that further studies into the diagnostic and therapeutic potential of miRNAs in equine OA are warranted.

## Data availability statement

The original contributions presented in the study are included in the article/Supplementary material, further inquiries can be directed to the corresponding author.

## Ethics statement

The study was approved by the Danish Animal Experiments Inspectorate (approval no. 2020-15-0201-00602 and 2017-15-0201-01314) as well as the local Ethical and Administrative body of the Department of Veterinary Clinical Sciences, University of Copenhagen (# 2020-016 and #2017-010). The study was conducted in accordance with the local legislation and institutional requirements.

## Author contributions

CA: Conceptualization, Formal analysis, Investigation, Methodology, Project administration, Writing – original draft, Writing – review & editing. MW: Conceptualization, Investigation, Methodology, Project administration, Writing – original draft, Writing – review & editing. LB: Conceptualization, Investigation, Methodology, Writing – original draft, Writing – review & editing. LCB: Conceptualization, Methodology, Supervision, Writing – original draft, Writing – review & editing. LV: Formal analysis, Writing – original draft, Writing – review & editing. EL-Å: Conceptualization, Writing – original draft, Writing – review & editing. BH: Data curation, Investigation, Visualization, Writing – original draft, Writing – review & editing. CL: Conceptualization, Methodology, Project administration, Supervision, Writing – original draft, Writing – review & editing. KS: Conceptualization, Data curation, Formal analysis, Investigation, Methodology, Project administration, Resources, Supervision, Writing – original draft, Writing – review & editing. SJ: Conceptualization, Formal analysis, Funding acquisition, Investigation, Methodology, Project administration, Supervision, Writing – original draft, Writing – review & editing.
