# Supplementary material for: Intraarticular treatment with integrin α10β1-selected mesenchymal stem cells affects microRNA expression in experimental post-traumatic osteoarthritis in horses
Source: Front Vet Sci. 2024 Mar 26;11:1374681. doi: 10.3389/fvets.2024.1374681 (PMC11002141; doi:10.3389/fvets.2024.1374681)
Supplement: Supplementary file 1 [file Table_1.DOCX]

**Supplementary table 1**

| **miRNA name** | **Forward** | **Reverse** |
| --- | --- | --- |
| let-7a | GCAGTGAGGTAGTAGGTTGT | GTCCAGTTTTTTTTTTTTTTTAACT |
| let-7c | GCAGTGAGGTAGTAGGTTGTA | GTCCAGTTTTTTTTTTTTTTTAACCA |
| let-7d-5p | GCAGAGAGGTAGTAGGTTGC | GGTCCAGTTTTTTTTTTTTTTTAACTATG |
| let-7d-5p | CAGAGAGGTAGTAGGTTGCAT | GGTCCAGTTTTTTTTTTTTTTTAACTATG |
| let-7e | CAGTGAGGTAGGAGGTTGT | GGTCCAGTTTTTTTTTTTTTTTAACTATAC |
| let-7f | CGCAGTGAGGTAGTAGATTG | GGTCCAGTTTTTTTTTTTTTTTAACTATAC |
| let-7g | CGCAGTGAGGTAGTAGTTTG | GTCCAGTTTTTTTTTTTTTTTAACTGT |
| miR-10a-5p | GCAGTACCCTGTAGATCCGA | GGTCCAGTTTTTTTTTTTTTTTACAAATTC |
| miR-10b-5p | CAGTACCCTGTAGAACCGA | GGTCCAGTTTTTTTTTTTTTTTCACA |
| miR-19a | GCAGTGTGCAAATCTATGC | GGTCCAGTTTTTTTTTTTTTTTCAG |
| miR-20a-5p | CGCAGTAAAGTGCTTATAGTG | GTCCAGTTTTTTTTTTTTTTTACCTG |
| miR-23a-3p | CATCACATTGCCAGGGAT | CGTCCAGTTTTTTTTTTTTTTTGGAA |
| miR-26a-5p | CGAGTTCAAGTAATCCAGGA | CCAGTTTTTTTTTTTTTTTAGCCTATC |
| miR-27a-3p | CAGTTCACAGTGGCTAAGA | CAGTTTTTTTTTTTTTTTGCGGAA |
| miR-27b-3p | CAGTTCACAGTGGCTAAGTTC | TCCAGTTTTTTTTTTTTTTTGCAGA |
| miR-28-3p | CGCAGCACTAGATTGTGAG | CCAGTTTTTTTTTTTTTTTCCAGGA |
| miR-28-5p | CAGAAGGAGCTCACAGTCT | GGTCCAGTTTTTTTTTTTTTTTCTCA |
| miR-29a | GCTAGCACCATCTGAAATCG | TCCAGTTTTTTTTTTTTTTTAACCGA |
| miR-29b | CAGTAGCACCATTTGAAATCAGT | GGTCCAGTTTTTTTTTTTTTTTAACACT |
| miR-30b-5p | GCAGTGTAAACATCCTACACTC | CCAGTTTTTTTTTTTTTTTAGCTGAG |
| miR-30c-5p | CAGTGTAAACATCCTACACTCTC | CCAGTTTTTTTTTTTTTTTGCTGAG |
| miR-30d | GTAAACATCCCCGACTGGA | CCAGTTTTTTTTTTTTTTTAGCTTCC |
| miR-98-5p | CGCAGTGAGGTAGTAAGTTGT | AGGTCCAGTTTTTTTTTTTTTTTAACA |
| miR-99a-5p | CAGAACCCGTAGATCCGA | TCCAGTTTTTTTTTTTTTTTCACAAGA |
| miR-101 | CGCAGTACAGTACTGTGAT | GGTCCAGTTTTTTTTTTTTTTTCAG |
| miR-101 | GCGCAGTACAGTACTGTGA | GGTCCAGTTTTTTTTTTTTTTTCAGTT |
| miR-122 | GTGGAGTGTGACAATGGTG | GTCCAGTTTTTTTTTTTTTTTACAAACA |
| miR-125a-3p | CAGGTGAGGTTCTTGGGA | TCCAGTTTTTTTTTTTTTTTGGCT |
| miR-125a-3p | GACAGGTGAGGTTCTTGG | GTTTTTTTTTTTTTTTGGCTCCCA |
| miR-125a-5p | CCCTGAGACCCTTTAACCT | GTCCAGTTTTTTTTTTTTTTTCACAG |
| miR-139-3p | CGCGGCCCTGTTG | GTCCAGTTTTTTTTTTTTTTTACTCCA |
| miR-139-5p | CTACAGTGCACGTGTCTC | GGTCCAGTTTTTTTTTTTTTTTCTG |
| miR-139-5p | CTACAGTGCACGTGTCTC | CCAGTTTTTTTTTTTTTTTCTGGAG |
| miR-140-3p | GTACCACAGGGTAGAACCA | GTCCAGTTTTTTTTTTTTTTTCCGT |
| miR-140-5p | CAGAGTGGTTTTACCCTATGG | GTCCAGTTTTTTTTTTTTTTTCTACC |
| miR-146a-5p | GCAGTGAGAACTGAATTCCA | GGTCCAGTTTTTTTTTTTTTTTAACC |
| miR-148a-3p | CAGTCAGTGCACTACAGAAC | GGTCCAGTTTTTTTTTTTTTTTACAAAG |
| miR-148a-3p | GCAGTCAGTGCACTACAG | GGTCCAGTTTTTTTTTTTTTTTACAAAG |
| miR-148b-3p | AGTCAGTGCATCACAGAAC | GGTCCAGTTTTTTTTTTTTTTTACAAAG |
| miR-148b-3p | GCAGTCAGTGCATCACAGA | GGTCCAGTTTTTTTTTTTTTTTACAAAG |
| miR-148b-5p | GCAGGAAGTTCTGTTATACACTC | CCAGTTTTTTTTTTTTTTTCCTGAG |
| miR-148b-5p | CGCAGGAAGTTCTGTTATACAC | CAGTTTTTTTTTTTTTTTCCTGAGTG |
| miR-150-5p | GTCTCCCAACCCTTGTAC | GTCCAGTTTTTTTTTTTTTTTCACTG |
| miR-151-5p | TCGAGGAGCTCACAGTC | GTCCAGTTTTTTTTTTTTTTTACTAGAC |
| miR-151-5p | TCGAGGAGCTCACAGTC | GGTCCAGTTTTTTTTTTTTTTTACTAGA |
| miR-155 | CGCAGTTAATGCTAATCGTGA | CCAGTTTTTTTTTTTTTTTACCCCTA |
| miR-155-5p | CGCAGTTAATGCTAATTGTGATAG | CCAGTTTTTTTTTTTTTTTCCCCTA |
| miR-182-5p | TTGGCAATGGTAGAACTCAC | GGTCCAGTTTTTTTTTTTTTTTCAG |
| miR-182-5p | AGTTTGGCAATGGTAGAACTC | CCAGTTTTTTTTTTTTTTTCAGTGTG |
| miR-183 | GCAGTATGGCACTGGTAGA | TCCAGTTTTTTTTTTTTTTTCAGTGA |
| miR-184 | CAGTGGACGGAGAACTGA | GTCCAGTTTTTTTTTTTTTTTACCCT |
| miR-186 | CAGCAAAGAATTCTCCTTTTGG | CCAGTTTTTTTTTTTTTTTAAGCCCA |
| miR-192 | GCAGCTGACCTATGAATTGAC | CAGTTTTTTTTTTTTTTTGGCTGTC |
| miR-195 | CGCAGTAGCAGCACAGA | TCCAGTTTTTTTTTTTTTTTGCCA |
| miR-196b-5p | CAGTTCACAGTGGCTAAGTTC | CAGTTTTTTTTTTTTTTTGCGGAA |
| miR-196b-5p | GCAGTTCACAGTGGCTAAG | CAGTTTTTTTTTTTTTTTGCGGAA |
| miR-199b-3p | GCAGTACAGTAGTCTGCAC | TCCAGTTTTTTTTTTTTTTTAACCAATG |
| miR-199b-5p | CCCAGTGTTCAGACTACCT | CCAGTTTTTTTTTTTTTTTGAACAGG |
| miR-200a | CAGTAACACTGTCTGGTAACG | CCAGTTTTTTTTTTTTTTTAACATCGT |
| miR-200b-3p | CGCAGTAATACTGCCTGGT | GGTCCAGTTTTTTTTTTTTTTTCATCA |
| miR-200b-3p | CGCAGTAATACTGCCTGGT | GGTCCAGTTTTTTTTTTTTTTTCATC |
| miR-200c | AGTAATACTGCCGGGTAATGA | GGTCCAGTTTTTTTTTTTTTTTCCA |
| miR-200c | AGTAATACTGCCGGGTAATGA | TCCAGTTTTTTTTTTTTTTTCCATCA |
| miR-214-3p | CAGCAGGCACAGACAG | CAGGTCCAGTTTTTTTTTTTTTTTACT |
| miR-214-3p | ACAGCAGGCACAGACA | CAGGTCCAGTTTTTTTTTTTTTTTACT |
| miR-215 | CGCAGATGACCTATGAATTGAC | GGTCCAGTTTTTTTTTTTTTTTGTC |
| miR-296-5p | GGCCCCCCCCAATC | GTCCAGTTTTTTTTTTTTTTTACAGGA |
| miR-329b-3p | CGCAGAACGAACCTGGT | GTCCAGTTTTTTTTTTTTTTTAAAAGAGG |
| miR-340-3p | GCAGTCCGTCTCAGTTAC | GTCCAGTTTTTTTTTTTTTTTGGCTA |
| miR-340-5p | CGCAGTTATAAAGCAATGAGAC | GTCCAGTTTTTTTTTTTTTTTAATCAGTC |
| miR-342-3p | GTCTCACACAGAAATCGCA | CCAGTTTTTTTTTTTTTTTACGGGT |
| miR-342-5p | CAGAGGGGTGCTATCTGT | TCCAGTTTTTTTTTTTTTTTCAATCAC |
| miR-374a-5p | AGCGCAGTTATAATACAACCTG | GGTCCAGTTTTTTTTTTTTTTTCACT |
| miR-378 | AGACTGGACTTGGAGTCAG | CAGTTTTTTTTTTTTTTTGCCTTCTG |
| miR-409-3p | GGAATGTTGCTCGGTGAAC | AGGTCCAGTTTTTTTTTTTTTTTAGG |
| miR-499-3p | AGAACATCACAGCAAGTCTG | CCAGTTTTTTTTTTTTTTTAGCACAG |
| miR-499-5p | GCAGTTAAGACTTGCAGTGA | GTCCAGTTTTTTTTTTTTTTTAAACATCA |
| miR-499-5p | GCAGTTAAGACTTGCAGTGA | GGTCCAGTTTTTTTTTTTTTTTAAACATC |
| miR-676 | GCCGTCCTAAGGTTGTTGA | GGTCCAGTTTTTTTTTTTTTTTAACTC |
| miR-872 | CGCAGAAGGTTACTTGTTAGTTC | GTCCAGTTTTTTTTTTTTTTTCCTGA |
| miR-1307 | GCGTGGCGTCGGT | TCCAGTTTTTTTTTTTTTTTCACGA |
| miR-1388-5p | AGGACTGTCCAACCTGAG | GGTCCAGTTTTTTTTTTTTTTTACCA |
| miR-1839 | GCAGAAGGTAGATAGAACAGGTC | GTCCAGTTTTTTTTTTTTTTTCAAGAC |
| miR-7177b | CAGTAAATGATCCCCTGGTG | GGTCCAGTTTTTTTTTTTTTTTCTAGG |
| miR-7177b | GAAATGGTCCCCTAGTGCT | GGTCCAGTTTTTTTTTTTTTTTCCA |
| miR-8992 | CGCGCTGCAGGCT | GGTCCAGTTTTTTTTTTTTTTTCCC |

A list of miRNAs and specific primer sequences.
